# A Case-Control Study of Titanium and Fluoroplastic Ventilation Tubes

**DOI:** 10.7759/cureus.32633

**Published:** 2022-12-17

**Authors:** Majidah S Alshammari, Sarah A AlOthman, Abdullah Sindi, Talal Al-Khatib

**Affiliations:** 1 Otolaryngology, Head and Neck Surgery, Faculty of Medicine, King Abdulaziz University, Jeddah, SAU; 2 Otorhinolaryngology, Head and Neck surgery, Faculty of Medicine, King Faisal Specialist Hospital and Research Center, Jeddah, SAU

**Keywords:** otorrhea, fluoroplastic, titanium, otitis media, blockage, ventilation tube, complications

## Abstract

Background

Tympanostomy ventilation tube (VT) insertion is one of the most common procedures performed in otorhinolaryngology. VTs have been proven to effectively manage otitis media (OM) with effusion (OME) and to improve the quality of life of children postoperatively. Although there are multiple types of VT shapes, materials, and sizes, few studies have investigated and compared the effects of titanium VT with those of VTs made of other materials. This study aimed to compare titanium VTs and the more commonly used fluoroplastic VTs in a retrospective, age-matched, case-control study. We studied the postoperative outcomes and rates of extrusion, infection, otorrhea, tube obstruction, and residual perforation.

Methodology

Medical records of patients who underwent myringotomy with VT insertion from January 2018 to December 2020 were reviewed. A total of 34 patients met the inclusion criteria, of whom 17 had undergone titanium VT insertion bilaterally (titanium group) and 17 had undergone fluoroplastic VT insertion bilaterally (control group). Both groups were followed up with regular postoperative examinations for 18 months.

Results

Postoperative complications were categorized as early and late complications. The most common early postoperative complication was early extrusion of VT (six months or less after insertion) (67.6%); this was documented most often in the titanium group. Other early postoperative complications included transient otorrhea (14.7%), tube blockage (8.8%), and recurrent acute otitis media (AOM) (occurring within one month from completion of therapy of AOM episode) (5.9%); these rates were similar in both groups. Late complications were not significantly variable between groups. Tympanic membrane retraction was the most common late complication (8.8%).

Conclusions

VT insertion is associated with the risk of complications with varying degrees. Although factors affecting the VT complication rates are multiple and various, these rates were not different between groups in this study. However, further studies including larger population samples are needed to statistically confirm these results and their generalizability.

## Introduction

Otitis media (OM) with effusion (OME) is defined as the presence of fluid in the middle ear without signs of acute infection [1]. As one of the most common diagnoses among young children, it affects more than 50% of children before the age of one year and more than 60% of children before the age of two years [2]. Furthermore, it affects approximately 90% of children before the age of five years [3]. OME is considered the leading cause of hearing loss among children in developed countries [4]. Approximately 75-90% of OME cases resolve spontaneously within three months [5]; however, approximately 25% of cases do not resolve and may be associated with hearing loss [6], delay in language development [7], balance (vestibular) problems, poor school performance, behavioral problems, ear discomfort, and recurrent acute otitis media (AOM), which negatively impact the quality of life [8]. Therefore, OME places a burden on the healthcare system [9]. As a result, tympanostomy ventilation tubes (VTs) have become one of the recommended treatments of choice for OME [10] because they have been proven to effectively manage OME and improve the quality of life of children with this condition [11].

VTs, which usually have a width of approximately one-twentieth of an inch, are inserted surgically in the tympanic membrane to ventilate the middle ear space [8]. Even though there are multiple different types of VT shapes, materials, and sizes, only a few studies have investigated and compared the effects and functions of different VT materials, specifically titanium [11]. The reported results have included various rates of extrusion, infection, and other side effects. Therefore, this study compared the results of titanium VT implantation and fluoroplastic VT insertion among children by performing a retrospective, age-matched, case-control study. We studied the postoperative outcomes and rates of extrusion, infections, otorrhea, tube obstruction, and residual perforation.

## Materials and methods

Population

Medical records of patients who underwent myringotomy with VT insertion for recurrent AOM or OME lasting for more than 90 days from January 2018 to December 2020 were reviewed. All patients who met the inclusion criteria and provided consent were included in the study.

Inclusion criteria

The inclusion criteria were age older than six months, diagnosis of unilateral or bilateral OME for three months or longer without resolution as evidenced by persistent type B (flat) tympanogram, and documented hearing loss.

Exclusion criteria

The exclusion criteria were age younger than six months, age older than 12 years, diagnosis of retraction-type ear disease (atelectasis or adhesive OM), complications such as AOM or barotrauma, and use of medications administered in the middle ear (for conditions such as sudden idiopathic sensorineural hearing loss or Meniere’s disease).

Procedure

All patients underwent myringotomy with VT insertion bilaterally. The procedure was performed in a routine manner under general anesthesia, with myringotomy performed in the anterior inferior quadrant. A Sheehy collar button titanium VT size 1.0 mm or a Shah fluoroplastic VT size 1.14 mm was inserted in the tympanic membrane.

Postoperative follow-up duration

Medical records indicated the follow-up results at two weeks and data obtained at three-month intervals for a duration of 12 months or until the VTs were extruded. Because the patients were followed up at three-month intervals, the exact time when the VTs were extruded was difficult to pinpoint; therefore, the extrusion time was set as the day of the follow-up visit for statistical and research purposes.

Medical records

Of the 34 patients enrolled in this study, three had some missing follow-up data. Therefore, a telephone interview was conducted to obtain the missing data.

Statistical analysis

The data obtained during the study were analyzed using SPSS Statistics for Windows version 23 (IBM Corp., Armonk, NY, USA). The Shapiro-Wilk test was performed to evaluate the normal distribution. The collected values are presented as the mean ± standard deviation and range for parametric data; they are presented as the frequency (%) for categorized data. Statistical comparisons were performed using the Kruskal-Wallis test for abnormally distributed parametric data and the Pearson chi-square test for categorized data. P-values of <0.05 were considered statistically significant.

## Results

A total of 34 patients met the inclusion criteria, with 17 (50%) male patients and 17 (50%) female patients. Fluoroplastic VT insertion was performed for 17 patients, and titanium VT insertion was performed for 17 patients. The mean age of all patients in this study was 6.12 years (range = 2-11 years). The difference in the ages of patients who received fluoroplastic VTs and those who received titanium VTs was insignificant (p = 0.171) (Table 1).

**Table 1:** Demographic characteristics of patients according to the type of tympanostomy ventilation tube. Data are expressed as mean ± standard deviation (range) or frequency (%) as appropriate. Comparisons between parametric parameters were performed using the Kruskal-Wallis test. Comparisons between categorized data were performed using the Pearson chi-square test.

Characteristics	Total (n = 34)	Fluoroplastic group (n = 17)	Titanium group (n = 17)	P-value
Age (years)	6.12 ± 2.48 (2.00–11.00)	6.71 ± 3.00 (2.00–11.00)	5.53 ± 1.74 (3.00–9.00)	0.171
Gender				0.500
Male	17 (50.0%)	9 (52.9%)	8 (47.1%)	
Female	17 (50.0%)	8 (47.1%)	9 (52.9%)	
Ears (n)	68	34	34	
Perioperative complications	None	None	None	
Child in daycare/school				0.360
Yes	12 (35.3%)	5 (29.4%)	7 (41.2%)	
No	22 (64.7%)	12 (70.6%)	12 (70.6%)	
Smoking in the home				0.500
Yes	5 (14.7%)	2 (11.8%)	3 (17.5%)	
No	29 (85.3%)	15 (88.2%)	14 (82.4%)	

During the perioperative period, no complications (falling VT in the middle ear, insufficient myringotomy, bleeding) were recognized. There were no reports of ossicular chain disruption, extended inflammatory illness such as labyrinthitis, or endocranial sequelae during surgery. The patients did not have any issues with general anesthesia.

The VTs were extruded spontaneously from 17 (100%) patients in the fluoroplastic group and 14 patients in the titanium group. The other three patients in the titanium group had no difficulties during the 12 months of follow-up, and their VTs were intentionally removed after 24 months. Nine patients (29.4% in the fluoroplastic group and 23.5% in the titanium group) underwent late tube extrusion. The fluoroplastic group had longer tube placement (mean = 15.31 ± 8.15 months; range = 6-36 months) than the titanium group (mean = 7.60 ± 6.10 months; range = 1-20 months) (p = 0.004) (Table 2).

**Table 2:** Early complications of otitis media according to the type of tympanostomy ventilation tube. Data are expressed as mean ± standard deviation (range) or frequency (%) as appropriate. Comparisons between parametric parameters were performed using the Kruskal-Wallis test. Comparisons between categorized data were performed using the Pearson chi-square test.

Complications	Total (n = 34)	Fluoroplastic group (n = 17)	Titanium group (n = 17)	P-value
Tube blockage	3 (8.8%)	2 (11.8%)	1 (5.9%)	0.500
Recurrent acute otitis media	2 (5.9%)	1 (5.9%)	1 (5.9%)	0.758
Transient otorrhea	5 (14.7%)	2 (11.8%)	3 (17.6%)	0.500
Early extrusion (six months or less)				0.226
No	9 (26.5%)	5 (29.4%)	4 (23.5%)	-
Yes	23 (67.6%)	11 (64.7%)	12 (70.6%)	-
I do not know	2 (5.9%)	1 (5.9%)	1 (5.9%)	-
Tube placement duration (months)	11.18 ± 7.64 (1–36)	15.31 ± 8.15 (6–36)	7.60 ± 6.10 (1–20)	0.004

Early postoperative complications included tube blockage in three (8.8%) patients and recurrent AOM (occurring within one month of completion of therapy of an AOM episode) [12] in two (5.9%) patients. Transient otorrhea, defined as occasional middle ear secretion more than three months after VT insertion, was documented for five (14.7%) patients. Tympanosclerosis, perforation, retraction of the tympanic membrane, and cholesteatoma comprised the functional and permanent structural sequelae observed after VT extraction (Table 2). Retraction of the tympanic membrane was the most common late postoperative complication (three patients, 8.8%), followed by permanent perforation (one patient, 2.9%), the need for a second tube (one patient, 2.9%), tympanosclerosis (one patient, 2.9%), and cholesteatoma [13] (one patient, 2.9%) (Table 3).

**Table 3:** Late complications of otitis media according to the type of tympanostomy ventilation tube.

Complications	Total (n = 34)	Fluoroplastic group (n = 17)	Titanium group (n = 17)	P-value
Permanent perforation	1 (2.9%)	1 (5.9%)	-	0.500
Second tube (repeat surgery) required	1 (2.9%)	-	1 (5.9%)	0.500
Tympanosclerosis	1 (2.9%)	-	1 (5.9%)	0.500
Retraction	3 (8.8%)	-	3 (17.6%)	0.114
Cholesteatoma	1 (2.9%)	-	1 (5.9%)	0.500

## Discussion

One of the most common therapeutic interventions for the treatment of multiple middle ear pathologies such as persistent middle ear effusion, recurrent middle ear infections (recurrent OM), or middle ear infections that persist after proper therapy is VT insertion [7].

The effects of VT treatment on the hearing and language of children have been studied. At least eight randomized controlled trials have studied the effects of VT on the hearing and language development of children 1.5-9 years of age. These studies involved follow-up periods of up to 10 years and reported the beneficial effects of VT on hearing and language development [12-19]. A systematic review by Hellström et al. [20] in 2011 showed that VT treatment had a beneficial impact on hearing, particularly for those with the most severe disability before VT insertion. Despite the fact that VT insertion has great benefits, it can also have unfavorable results [20].

Therefore, as with any other medical procedure or intervention, the benefits should be weighed against the associated risks. The risks associated with VT insertion can occur intraoperatively or immediately postoperatively. Perioperative risks include those associated with general anesthesia, such as laryngospasm and bronchospasm, which occur more commonly in children than in adults [21]. Intraoperative complications include VT misplacement in the middle ear, insufficient myringotomy, bleeding, ossicular chain disruption, extended inflammatory illness such as labyrinthitis, and endocranial sequelae. During our study, none of the included patients reported any issues with general anesthesia or other preoperative complications. Other early and late postoperative issues are associated with VT insertion as well [20]. During our study, we did not find any reports of perioperative incidents in the medical records, which is consistent with previously published data [22].

The early and late postoperative issues associated with VT insertion vary according to the surgery techniques used, the frequency of postoperative examinations, and the type of VT used [23]. Based on the literature, we used a period of 18 months as the cutoff point for early and late surgical complications. To study the efficacy of titanium VTs, we compared their outcomes with those of fluoroplastic Shah VTs inserted in a group of control patients treated at our facility. Analysis of our data could not detect a significant difference in the ages of the patients in the fluoroplastic and titanium groups. Furthermore, otorrhea was the most common complication that occurred with VTs (up to 50% of patients) [24]. These results are consistent with those of previous studies of complications associated with VTs that concluded that two out of every three children with VTs develop otorrhea [25].

However, when the rates of early complications associated with titanium VTs and fluoroplastic VTs were compared in our study, the data analysis could not detect a significant difference in the otorrhea rates of the two groups. Early extrusion of the VTs was the most common early postoperative complication during our study. The extrusion rate was the only early complication that was statistically significant. The rate of spontaneous extrusion was higher for the fluoroplastic VTs, and the duration of VT placement was longer for the titanium VTs. There are different types of VTs that are designed to last for various lengths of time. Titanium and fluoroplastic VTs are both labeled for short-term use and should last 12 to 18 months.

Premature or early VT extrusion was defined as extrusion of the VTs at six months or earlier after insertion. The causes of early extrusion are variable. Iatrogenic causes, including wrongfully placed or executed myringotomies, which should be avoided, can lead to early extrusion. Early extrusion can also be attributed to an exaggerated response to a foreign body [26].

A retrospective study of 478 children that compared the early postoperative complications of different types of VTs reported that iatrogenic causes and inadequate insertion were the major causes of early extrusion of different VT types; furthermore, all patients who required early extrusion had postoperative otorrhea [26]. Additionally, Handler et al. compared titanium VTs to other types of VTs and found no statistically significant differences in the early complications associated with titanium VTs and silicone VTs [27]. Alternatively, late complications were not as common as early complications. The most common late complication observed in our study was tympanic membrane retraction. The most serious complication observed was cholesteatoma; fortunately, it was observed in only one patient. Cholesteatoma is a rare complication of VT insertion, with a risk of 0.5% [13]. It has been reported that patients who develop cholesteatoma usually present with persistent otorrhea first [13].

Cholesteatoma related to VT insertion is considered a VT complication if it is found behind a tympanic membrane perforation that developed at the site of VT insertion or at the intact tympanic membrane near the VT insertion site (i.e., in the mesotympanum or hypotympanum) [13]. We encountered the case of a five-year-old female patient who required VT insertion twice for OME that did not resolve after three months of observation and supportive medical therapy. She was followed up routinely after surgery. During follow-up, she developed persistent otorrhea. After VT extraction, she had a retraction pocket in the same area where previous surgeries had been performed.

The retraction pocket was suspicious; therefore, an investigation was performed and a small cholesteatoma in the mesotympanum was detected behind the intact tympanic membrane. The cholesteatoma was treated with surgical intervention and a follow-up was performed. Finally, the rates of late complications associated with titanium VTs and those associated with standard fluoroplastic VTs were not statistically significantly different.

Limitations

This research was conducted in a single research facility using data of patients known and followed up in the center. Hence, the main limitation of our study is the number of patients who had titanium VT insertion in our institute compared to the VTs of other materials.

It could be to an extent due to the high cost of titanium VTs compared to fluoroplastic tubes, and the lack of evidence in the literature to support the use of titanium tubes over other types.

The studies did not specify the type and size of fluoroplastic tubes as there are many variations that may affect extrusion rate and tympanic membrane perforation, further studies are needed to include other VT materials and types. In addition, some of the complications reported in our population relied mainly on self-reported history, rather than objective clinical methods. Therefore, larger sample sizes, as well as the inclusion of other VT materials, will be necessary to better understand the full effect and complications of VT material on the complication rate.

## Conclusions

As with any other surgical intervention, the use of VTs when indicated is associated with the risk of complications. The factors affecting complication rates are multiple and various. Analysis of the small population group included in this study could not detect a statistically significant difference in the complication rates, including those of early complications such as otorrhea and late complications, when different VT materials are used. Further research and studies including larger population samples are needed on the subject to conclude results applicable to the general population.
